# Ultra-long silver nanowires prepared *via* hydrothermal synthesis enable efficient transparent heaters†

**DOI:** 10.1039/d2na00560c

**Published:** 2022-08-29

**Authors:** Fevzihan Basarir, Swarnalok De, Hamidreza Daghigh Shirazi, Jaana Vapaavuori

**Affiliations:** Department of Chemistry and Materials Science, Aalto University P.O. Box 16100 FI-00076 Aalto Finland jaana.vapaavuori@aalto.fi

## Abstract

Ultra-long silver nanowires (AgNWs) with an aspect ratio of >2000 were prepared by the hydrothermal synthesis method. The influence of reaction time (4–32 h), reaction temperature (150–180 °C), polyvinylpyrrolidone (PVP) molecular weight (10 000–1 300 000 g mol^−1^), PVP concentration (50–125 mM), glucose concentration (5.6–22.4 mM) and CuCl_2_ concentration (2–20 μM) on the AgNW length was investigated systematically. The optimum conditions provided nanowires with an average diameter of 207 nm, an average length of 234 μm and a maximum length of 397 μm. Finally, a AgNW electrode was prepared on a glass substrate and used in transparent heater application. The transparent heater enabled outstanding heat-generating properties, reaching >200 °C within 70 s with an applied voltage of 5 V. Our results demonstrate how increasing the aspect ratio of ultra-long AgNWs is beneficial for both optical and electronic applications in terms of increased transmission and a more efficient Joule effect in the heater application. In addition, our results show that AgNWs with different lengths can be simply obtained by tuning synthesis parameters.

## Abstract 2

Ultra-long silver nanowires (AgNWs) with an aspect ratio of >2000 were prepared by the hydrothermal synthesis method.

## Graphical abstract

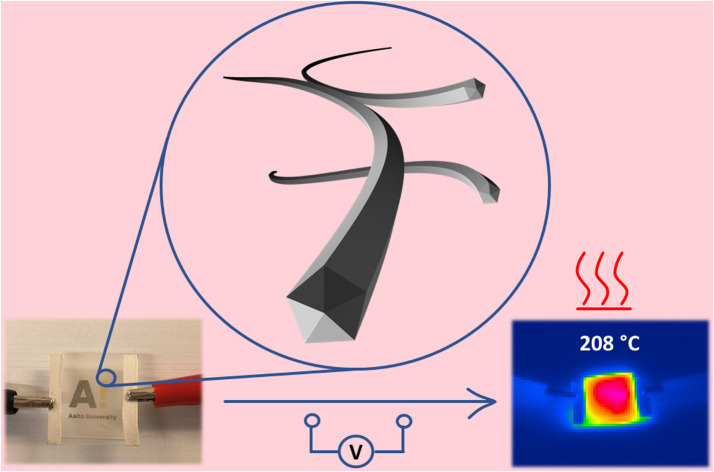

## Introduction

Owing to their ultra-high electrical conductivity, large aspect ratio and outstanding flexibility, silver nanowires (AgNWs) have been widely utilized in transparent electronics, such as transparent conductive films,^1,2^ organic photovoltaics (OPVs),^3–5^ organic light emitting diodes (OLEDs),^6,7^ touch screens,^8,9^ and transparent heaters.^10,11^ Besides, recently, utilization of AgNWs has expanded to numerous applications including flexible fuel cells,^12–14^ stretchable energy harvesters,^15^ flexible supercapacitors,^16^ flexible sensors,^17^ electronic skins,^18^ soft actuators,^19,20^ and air filters.^21,22^ High optical transmission and/or low sheet resistance are required to increase the performance of these devices, which pinpoints the need for the preparation of ultra-long AgNWs. In addition, use of longer nanowires may reduce the consumption of silver sources, which in turn may enhance sustainability.

Since the first introduction of polyol synthesis by the Xia group, it has been the most commonly employed method to synthesize AgNWs.^23^ In a typical synthesis route, ethylene glycol (EG) is used as both solvent and a reducing agent, while poly(vinylpyrrolidone) (PVP) is utilized as a capping agent, and silver nitrate (AgNO_3_) is used as the silver source providing AgNWs with a length of ∼10–20 μm.^24^ Consequently, several approaches have been developed to increase the length of the resulting AgNWs by adding 6-chlorohexylzinc bromide (∼40 μm),^25^ Cu^2+^ ions (∼49 μm),^26^ cocamidopropyl betaine (∼120 μm),^27^ Cr^3+^ ions (160 μm),^28^ Fe^3+^ (230 μm)^29^ or ascorbic acid (∼270 μm)^30^ to the reaction solution. In addition, Jiu *et al.*^31^ demonstrated the beneficial effect of stirring speed (∼100 μm), whereas Araki *et al.*^32^ investigated the influence of both temperature and stirring speed (∼230 μm) on the AgNW length. Additionally, propylene glycol^33^ (∼130 μm) or a propylene glycol/ethylene glycol mixture (∼77 μm)^34^ was used as solvent instead of ethylene glycol to increase the length of AgNWs. Besides, Lee *et al.*^24^ introduced successive multistep growth to obtain AgNWs with a maximum length of ∼280 μm. However, a very accurately controlled reaction setup is needed in polyol synthesis since the reaction is very sensitive to temperature, stirring speed and the injection rate of the precursors.

In comparison, the solvothermal synthesis method, which has a similar mechanism and uses the same precursors, has been investigated as an alternative to polyol synthesis owing to its simplicity, low-cost and ease of control. For instance, Liu *et al.*^35^ reported AgNWs with ∼10–20 μm while Zhang *et al.*,^36^ Chen *et al.*,^37^ Fang *et al.*,^38^ Xue *et al.*^39^ and Zhang *et al.*^40^ reported AgNWs with lengths of ∼50–250, ∼8–30 μm, ∼6–86 μm, ∼60–190 and ∼95–215 μm, respectively. Similar to the polyol method as discussed above, the impact of PVP MW,^39^ solvent type,^37^ PVP/AgNO_3_ molar ratio^35^ and salt type^36^ on the AgNW was examined individually. Despite its advantages, the maximum length of the AgNWs produced with the solvothermal method still remains below 300 μm.^35–40^

Recently, the hydrothermal synthesis method, which replaces ethylene glycol as solvent with water, was introduced.^41^ Interestingly, this approach generated AgNWs with maximum lengths of more than 300 μm in a single pot. However, despite the promising initial outcomes, to date, no systematic work has been carried out to increase the nanowire length, and the synthesis mechanism has not been fully established.

In this study, therefore, the preparation of ultra-long AgNWs (∼400 μm) by investigating the influence of synthesis parameters on the nanowire length was demonstrated. Differentiating the effects of different parameters on the synthesis results is expected to contribute towards establishing the synthesis mechanism. We have revealed that every parameter has an effect on the AgNW length and indicated that reaction temperature, PVP MW and concentration are the most significant ones. In addition, a transparent heater was fabricated using the AgNWs synthesized under the optimum conditions. This type of systematic pursuit of ultra-long AgNWs may contribute to increased performance with reduced silver consumption in versatile optoelectronic devices.

## Experimental

### Materials

Silver nitrate (AgNO_3_, ACS reagent, ≥99.0%), D+ glucose (ACS reagent), ethylene glycol (EG, anhydrous, 99.8%), copper(ii) chloride (CuCl_2_, 97%), sodium chloride (NaCl, ACS reagent, ≥99.0%), iron(iii) chloride (FeCl_3_, reagent grade, 97%) and polyvinylpyrrolidone (PVP) with average molecular weights of 10 000 (10k), 55 000 (55k), 360 000 (360k) and 1 300 000 (1300k) were purchased from Sigma-Aldrich. Deionized water (DI, 18.2 MΩ) was produced by a Millipore-Q system.

### Synthesis of silver nanowires

Silver nanowires (AgNWs) were synthesized by a facile hydrothermal synthesis method. In a typical procedure, separate solutions of AgNO_3_ (0.170 g, 5 ml), D+ glucose (0.06 g, 5 ml) and PVP-360k (0.167 g, 10 ml) were prepared at RT *via* dissolving and mixing vigorously in DI water. Then, D+ glucose solution was added to PVP solution and stirred for 5 min, followed by adding AgNO_3_ solution and stirring for an additional 5 min. Next, 100 μl of CuCl_2_ (4 mM in EG) was injected into the solution. The prepared mixture was transferred to a 50 ml Teflon lined stainless steel autoclave and heated in an oven at 160 °C for 24 h. Finally, the autoclave was naturally cooled down to RT.

Reaction time (4–32 h), reaction temperature (150–180 °C), PVP MW (10k–1300k), PVP concentration (50–125 mM), glucose concentration (5.6–22.4 mM), and CuCl_2_ concentration (2–20 μM) were evaluated as the essential parameters and in each reaction only one parameter was changed. Then, the influence of the parameter change on the AgNW length was investigated. Meanwhile, the silver concentration was kept constant (50 mM) in all the reactions. Besides, the effect of salt type was also examined with NaCl and FeCl_3_. The reaction details are summarized in Table S1.†

### Purification of nanowires

The AgNWs were purified by a modified procedure of a selective precipitation method.^42^ Briefly, after the synthesis, the brown color supernatant was discarded with a plastic pipette and the olive-green color precipitate was dissolved in 10 ml of DI water, followed by centrifugation at 2000 rpm for 20 min. The yellow color supernatant composed mainly of silver nanoparticles was removed with a pipette. The precipitated AgNWs were redispersed in 10 ml of water and 40 ml of acetone was added slowly and the AgNWs were allowed to settle down for 10 min. The supernatant was again removed with a pipette. This procedure was repeated 3–5 times until the supernatant became clear. The final product was dispersed in ethanol (5 mg ml^−1^) for further use.

### Fabrication of transparent heater samples

Prior to coating, the glass substrates (2.5 cm × 2.5 cm) were washed with excessive ethanol and dried under an air flow. Then, they were treated with a UV/ozone cleaner (Bioforce Nanosciences) for 30 min. Afterwards, AgNW solution was drop coated on the surface and the solvent was evaporated at RT, followed by annealing in a furnace at 250 °C for 30 min. The performance of the transparent heater samples was evaluated by using an infrared thermal camera (PIC UC 180, InfraTec). The AgNW films were heated by applying DC voltage at the silver contacts using a power supply (S-LS-85, Stamos).

### Characterization

The absorption spectra of the AgNWs in ethanol were recorded with a UV-Vis spectrometer (Shimadzu UV-2600) at RT. The morphology and microstructure of the nanowires were characterized by scanning electron microscopy (SEM, Tescan Mira3) and transmission electron microscopy (TEM, JEOL JEM-2800). The length and diameter of the AgNWs were evaluated with ImageJ software by measuring at least 100 nanowires and calculating a simple average value. X-ray reflectometry (XRR, PANalytical X'Pert) was utilized to identify the crystal structure of the nanowires.

## Results and discussion

### Characterization of silver nanowires

**Fig. 1:**
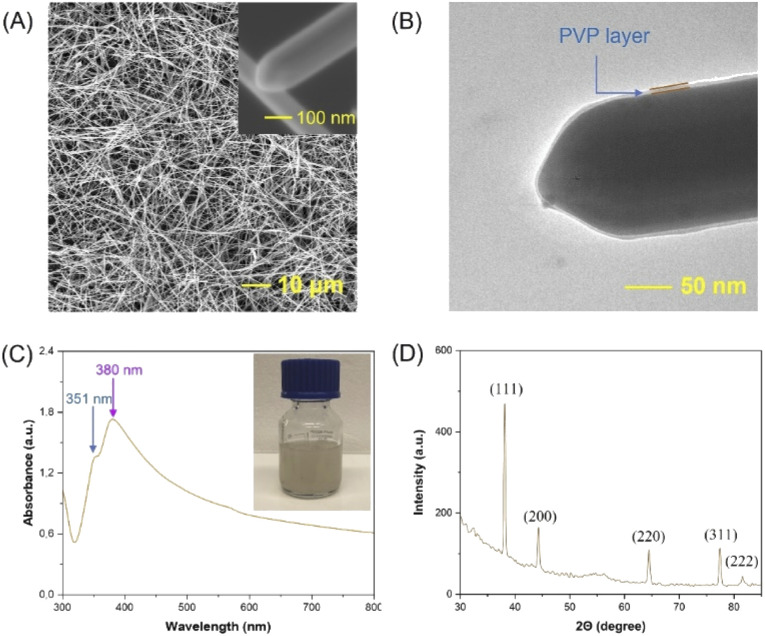
SEM (A), TEM (B), UV-Vis (C) and XRR (D) characterization results of the AgNWs.

The characterization of the AgNWs synthesized by a typical procedure (details given in the Experimental part) is reported in this subsection to lay the foundations for modifications of the synthesis procedure presented in subsequent subsections. Fig. 1A exhibits a SEM image showing a high aspect ratio and long silver nanowires (AgNWs) with a uniform diameter of ∼100–300 nm. The close-up SEM (inset of Fig. 1A) and TEM images (Fig. 1B) clearly demonstrate the 5-fold-twinned structure and rounded end shape of the AgNWs with a ∼3 nm PVP layer. In addition, the UV-vis absorption spectrum shows (Fig. 1C) absorption peaks at 351 and 380 nm, which could be attributed to the longitudinal and transverse plasmon mode of the nanowires,^35,41^ respectively, while the inset displays the purified AgNWs dispersed in ethanol (white-gray color).^24^Fig. 1D illustrates the XRD pattern of AgNWs, which could be indexed to face-centered cubic^41^ and it is notable that the intensity ratio of the reflection at (111) is higher than that at the others, indicating that (111) is the preferred orientation for the AgNWs. Based on TEM analysis, the lattice spacing of AgNWs was evaluated to be 0.24 nm, which is consistent with the ‘*d*’ value of the (111) plane of face-centered cubic silver (Fig. S1A†).^30^ The electron diffraction patterns, which are consistent with the XRR results (Fig. 1D), are illustrated in Fig. S1B.†

### Effect of reaction time

**Fig. 2:**
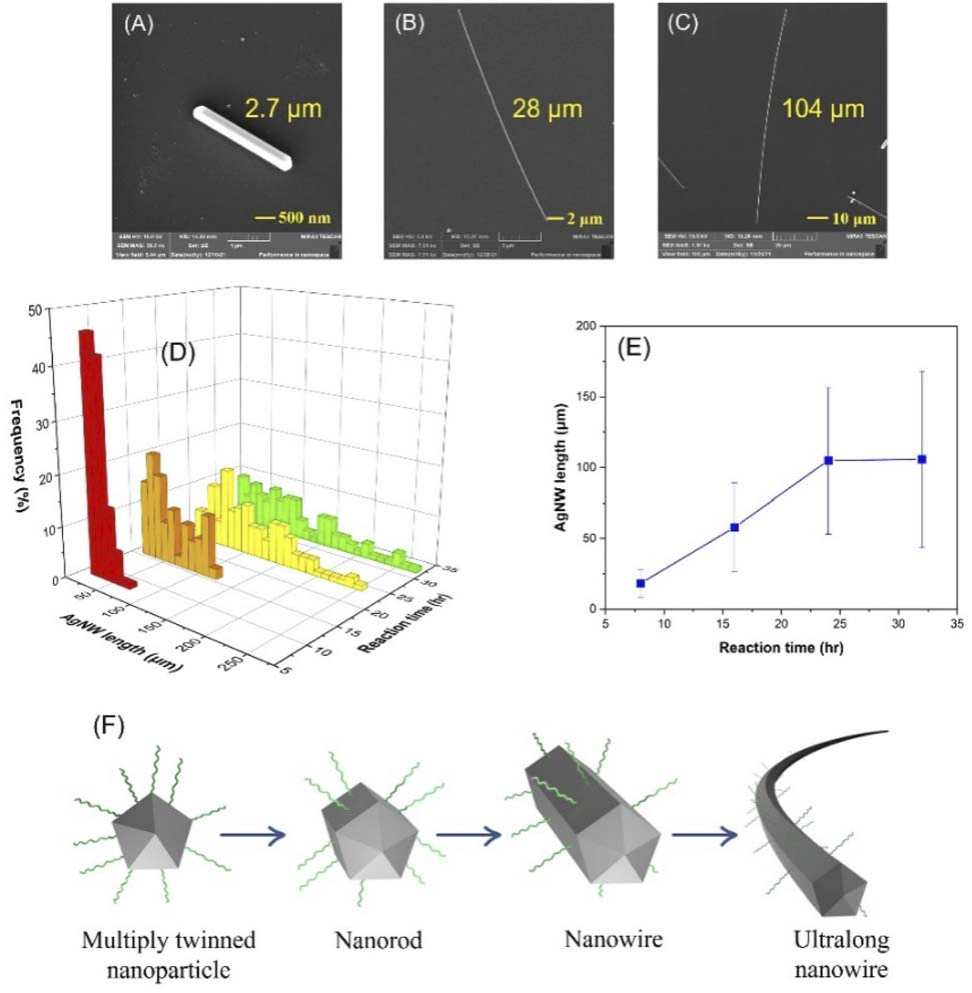
SEM images illustrating the effect of reaction time 4 h (A), 8 h and (B), 24 h (C) and length distribution of the AgNWs (D) and average size of the AgNWs (E) with respect to reaction time and (F) schematic illustration of ultralong nanowire formation.

The effect of reaction time was investigated by characterizing the AgNW length by SEM. As shown in Fig. 2A, a 4 h reaction provided only silver nanorods with a 5-fold-twinned structure and extending the reaction time yielded longer nanowires with no change in the diameter (Fig. 2B and C). The length distribution of the AgNWs with respect to time is demonstrated in Fig. 2D and the average length of the nanowires was found to be 18, 57, 105 and 106 μm for 8, 16, 24 and 32 hours (Fig. 2E), respectively, suggesting the evident increase in AgNW length as a function of increased reaction time. Additionally, the 2-D plot of the AgNW length distribution with respect to reaction time is displayed in Fig. S2.† This could be explained by the selectively passivating the {100} side surfaces with PVP, which in turn led to addition of silver atoms mainly to the {111} faceted ends.^43^ The formation of an ultralong nanowire is schematically illustrated in Fig. 2F. However, extending the reaction time beyond 24 h has no significant influence on the AgNW length, which could be explained by the consumption of all Ag^+^ ions in the reaction medium. It is notable that the required reaction time for the growth of nanowires is higher than that in the polyol (2–3 h)^43,44^ and solvothermal methods (3–12 h).^35,39,40^ We believe that this can be explained by two factors: (1) glucose is a milder reducing agent than ethylene glycol, which slows the reaction rate and (2) AgCl is less soluble in water than in ethylene glycol thus reducing the reaction speed.

### Effect of reaction temperature

**Fig. 3:**
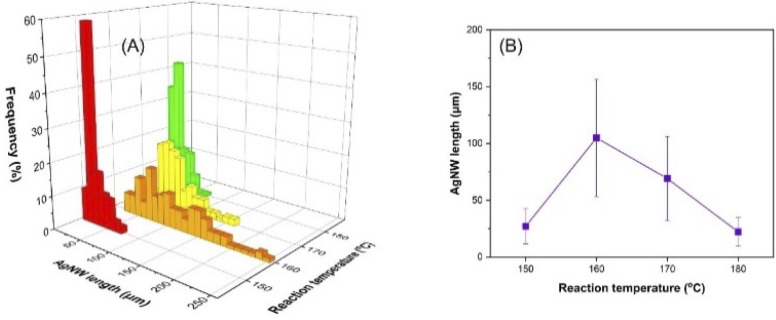
Influence of reaction temperature on the AgNW length distribution (A) and the average length of the nanowires (B).

A reaction temperature of 140 °C provided only silver nanoparticles while nanowires could be obtained at temperatures ranging from 150 to 180 °C (Fig. S3†). The length distribution of the AgNWs with respect to temperature is demonstrated in Fig. 3A and S4.† Initially, the average length of the AgNWs increased from 27 (150 °C) to 105 μm (160 °C) and then decreased to 69 (170 °C) and 22 μm (180 °C), as shown in Fig. 3B. As reported earlier, the reduction of the hydroxyl groups to aldehyde needs high temperature.^44^ Therefore, the initial increase of the AgNW length with temperature can be attributed to two factors: (1) increased number of electrons in the medium by the reduction of more glucose molecules and (2) increased reaction kinetics.

However, the later decrease in the nanowire length with increasing temperature can be explained by the carbonization of glucose molecules at higher temperature, which in turn leads to the formation of fewer electrons in the reaction medium. A similar trend was observed by Hemmati *et al.*, who reported formation of nanoparticles and nanowires at 140 °C and 150–170 °C by the polyol method, respectively.^44^ It is worth noting that the particles formed at 140 °C (Fig. S2A†) have nano/micron sized irregular shapes, which indicates the lack of a 5-fold-twinned structure. Thus, these results allow us to conclude that 150 °C is the critical temperature to form the specific crystalline faces, required for the growth of AgNWs.^45^

### Effect of PVP molecular weight

**Fig. 4:**
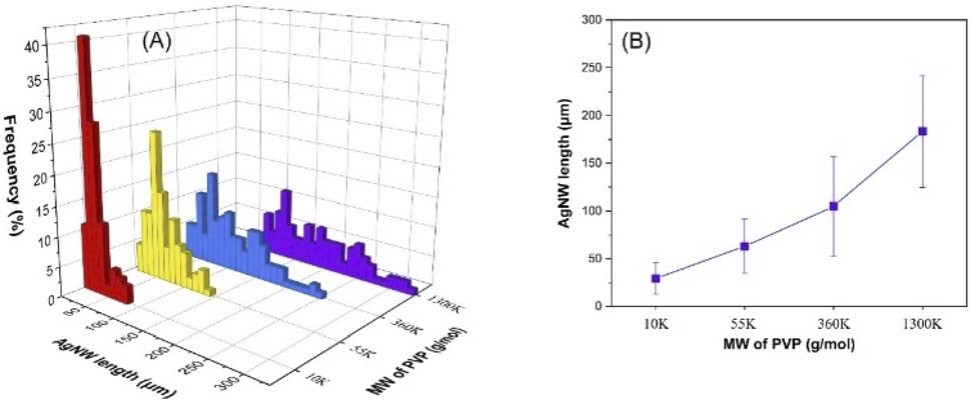
Influence of PVP MW on the AgNW length distribution (A) and the average length of the nanowires (B).

As demonstrated in Fig. 4 and S5,† the molecular weight of PVP has a significant effect on the AgNW length. Molecular weights of 10k, 55k, 360k and 1300k provided average lengths of 29, 63, 105 and 183 μm, respectively; however, no major influence on the diameter of the nanowires was observed. It is essential to note that the maximum length reached was 327 μm with the 1300k sample and the corresponding SEM images of the nanowires are shown in Fig. S6.† As mentioned above, PVP controls the growth of the nanowires *via* adsorbing on the {100} side surfaces of the silver seeds and allowing the Ag ions to deposit on the {111} faceted ends.^43^ Previously, the influence of PVP MW on the nanowire morphology in polyol and solvothermal methods was demonstrated by Hemmati *et al.*^44^ and Xue *et al.*,^39^ respectively. Both studies reported that the PVP MW has a great influence on both the adsorption strength of PVP on the side surface and the steric effect; however, contradictory results were shown. Without considering the diameter and length of the nanowires, Hemmati *et al.* found that a high MW led to a strong steric effect and weak adsorption on the {100} side surfaces, which resulted in the growth of other nanostructures.^44^ On the other hand, Xue *et al.* explored that a higher MW provided stronger chemical adsorption and a larger steric effect,^39^ which resulted in longer and thicker nanowires. In addition, formation of nanoparticles and nanorods was solely demonstrated with 10k PVP, which was attributed to very weak chemical adsorption and thus weak passivation of {100} side surface. However, in our work, AgNW formation was achieved already when employing 10k PVP, and longer nanowires were obtained with a higher MW of PVP with negligible change in the diameter. This can be attributed to the different solubilities of PVP in water and ethylene glycol, which in turn lead to different conformations of PVP in the reaction medium. We experimentally observed that water is a better solvent for PVP than ethylene glycol, and hence, we believe that PVP chains in ethylene glycol and water show coil-like and elongated conformation, respectively. Therefore PVP chains in the hydrothermal reaction medium can easily cover the {100} side surface and demonstrate a lower steric effect, due to the possible elongated conformation.

### Effect of PVP concentration

**Fig. 5:**
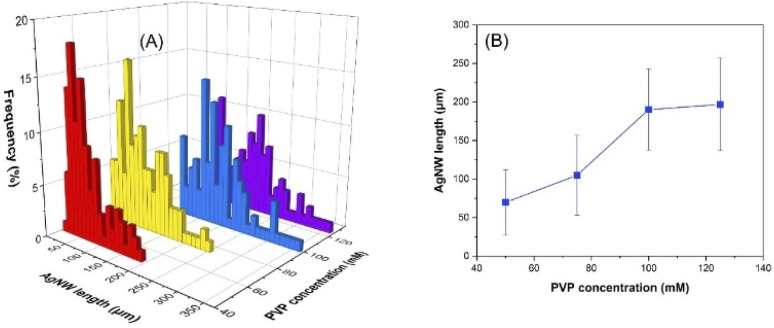
Influence of PVP concentration on the AgNW length distribution (A) and the average length of the nanowires (B).

Fig. 5 and S7† exhibit the length distribution and average length of the AgNWs synthesized with 50, 75, 100 and 125 mM PVP (360k), which corresponds to a PVP/AgNO_3_ molar ratio of 1, 1.5, 2 and 2.5, respectively. The corresponding SEM images of the nanowires are shown in Fig. S8.† With increasing the molar ratio, average lengths of 70, 105, 190 and 197 μm were achieved. It is worth noting that with a molar ratio higher than 1.5, a nanowire length longer than 350 μm could be obtained. The higher PVP/AgNO_3_ molar ratio in the reaction medium provides a greater number of PVP chains that will passivate the AgNW {100} side surface, which leads to longer nanowires. By the polyol method, Hemmati *et al.* indicated the formation of AgNWs with PVP/AgNO_3_ molar ratios of 0.78 and 1.56. However, unlike in our work, when the molar ratio was increased to 3.12, mainly nanoparticles were obtained.^44^ This could also be explained by the low solubility of PVP in ethylene glycol.

### Effect of glucose concentration

**Fig. 6:**
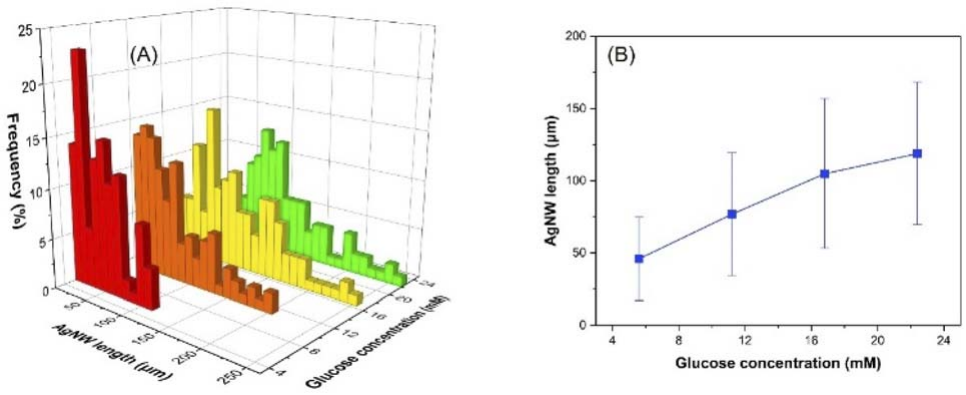
Influence of glucose concentration on the AgNW length distribution (A) and the average length of the nanowires (B).

Glucose is known as a green and mild reducing agent, and Fig. 6 and S9† show the length distribution and average length of the nanowires synthesized with 5.6, 11.2, 16.8 and 22.4 mM glucose. The increasing glucose amount provided average nanowire lengths of 46, 77, 105 and 119 μm. On the other hand, when 2.8 mM glucose was utilized, only nanoparticles could be obtained, demonstrating the lack of electrons for further growth of nanowires (Fig. S10†). It is worth noting that the increased concentration of glucose provides more electrons delivered to the reaction medium, resulting in more reduced silver ions and thus, anisotropic growth of longer nanowires.

### Effect of CuCl_2_ amount

**Fig. 7:**
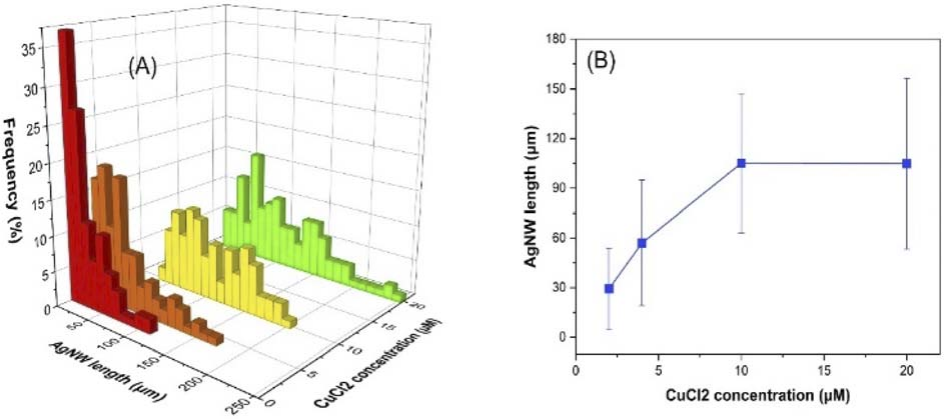
Influence of CuCl_2_ concentration on the AgNW length distribution (A) and the average length of the nanowires (B).

Cu(ii) is reduced to Cu(i) by the electrons delivered by glucose and Cl^−^ decreases the concentration of free silver ions in the reaction medium *via* forming AgCl. Due to its lower solubility, AgCl releases silver ions gradually into the reaction medium to form AgNWs. The adsorbed atomic oxygen was reported to block the reactive sites of the {111} faceted ends and Cu(i) scavenges the atomic oxygen, supporting the formation of longer nanowires.^43^ As the injected CuCl_2_ concentration increased, the average AgNW length increased to 29, 57 and 105 μm at 2, 4 and 10 μM, respectively, and then levelled off at higher concentrations (20 μM) (Fig. 7 and S11†). Doubling or halving the Cu^2+^ concentration (19.9 μM) in the previous report resulted in irregularly shaped Ag nanostructures.^43^ However, it is notable that AgNWs are formed in our work even with increasing or decreasing the Cu^2+^ concentration.

### Effect of salt type

**Fig. 8:**
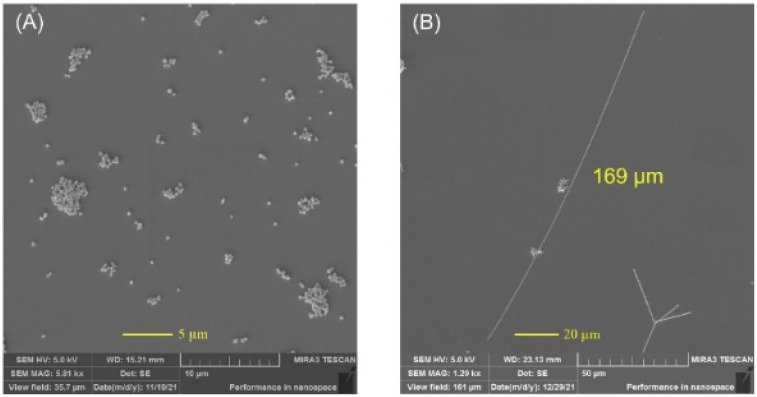
Influence of salt type (A) NaCl and (B) FeCl_3_.

Keeping all the other reaction parameters constant, the effects of NaCl and FeCl_3_ instead of CuCl_2_ were examined. As illustrated in Fig. 8A, only nanoparticles were formed when NaCl (20 μM) was used as salt. However, FeCl_3_ produced similar quality nanowires to CuCl_2_ (Fig. 8B). This could be explained by the atomic oxygen scavenging power of the Cu^2+^ and Fe^3+^ from the {111} faceted ends of the AgNWs. It was found that utilization of FeCl_3_ does not have a major effect on the nanowire length but it dramatically reduced the AgNW diameter to ∼40–230 nm, which is in good agreement with that in the previous report.^29^ The decreased diameter will mainly decrease the haziness of the thin films, which may be beneficial in optoelectronic applications. Besides, the morphology of the by-products of the AgNWs obtained with CuCl_2_ and FeCl_3_ is compared in Fig. S6.† It is notable that the by-product of synthesis with CuCl_2_ is composed of mainly irregularly shaped nanostructures and a small number of nanocubes and nanotriangles (Fig. S12A†). However, synthesis with FeCl_3_ resulted in by-products including nanorods and multiply twinned nanoparticles (Fig. S12B†).

To find evidence for interpreting the obtained results, we refer to standard reduction potentials of Na^+^ (−2.71 V), Cu^2+^ (+0.16 V) and Fe^3+^ (+0.77 V), as shown in Table S2,† demonstrating that reduction of Cu^2+^ and Fe^3+^ is a spontaneous reaction while that of Na^+^ is non-spontaneous. Therefore, Na^+^ ions cannot be reduced in the reaction medium, which in turn yielded only nanoparticles. On the other hand, compared to Cu^2+^, reduction of Fe^3+^ is more favorable, indicating that it is faster in scavenging the adsorbed oxygen atoms. Here, we hypothesize that the rate of oxygen scavenging and concentration of Cl^−^ ions are very important particularly in the early stages of the reaction. We believe that if the oxygen scavenging is faster, the probability of multiply twinned nanoparticle formation is higher and the nanoparticle diameter is smaller. In addition, the presence of more Cl^−^ ions in the medium leads to the formation of more AgCl, which in turn further slows the reaction rate and provides more multiply twinned nanoparticles.

On the other hand, it is crucial to mention that ultrasonication of AgNO_3_ solution (1–15 min) before placing in the reactor was tried but no influence on the nanowire length was observed.

### Synthesis under the optimum conditions

**Fig. 9:**
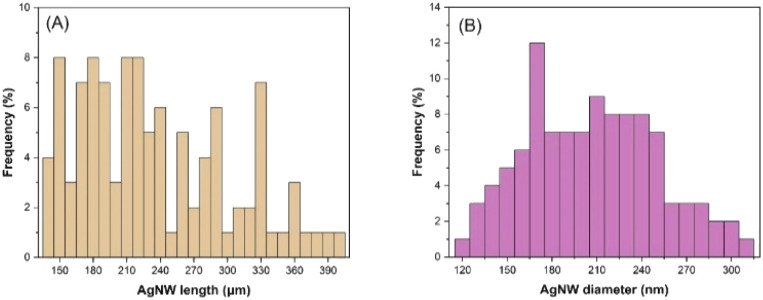
Length (A) and diameter (B) distribution of the AgNWs synthesized under optimum conditions.

The maximum nanowire lengths were obtained with a reaction time, reaction temperature, PVP MW, PVP concentration, glucose concentration and CuCl_2_ concentration of 24 h, 160 °C, 1300k, 125 mM, 22.4 mM and 20 μM, respectively. The length and diameter distribution of the nanowires synthesized under optimum conditions is shown in Fig. 9, which provided an average length and diameter of 234 μm and 207 nm. It is worth noting that the maximum length of 397 μm was reached under the optimum conditions, demonstrating an aspect ratio of >1000. On the other hand, when FeCl_3_ was used instead of CuCl_2_ under the optimum conditions, an average diameter of 131 nm was achieved without a significant change in the AgNW length, which resulted in an aspect ratio of >2000.

### Transparent heater application

**Fig. 10:**
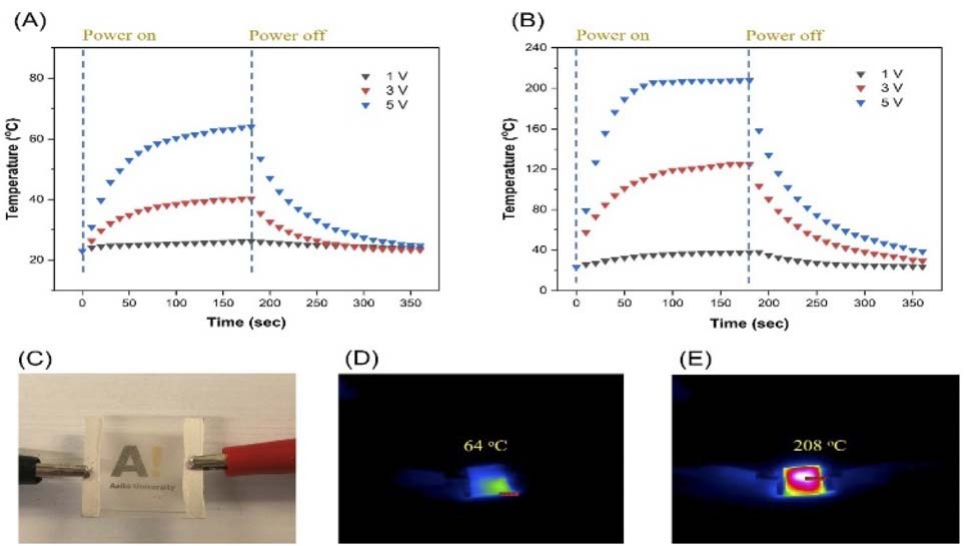
Time-dependent temperature profile of AgNW films (A) shorter nanowires and (B) longer nanowires, (C) macro photograph of the AgNW film based thermal heater, thermal camera images of the AgNW film at 5 V (D) shorter nanowires and (E) longer nanowires.

Transparent heaters were prepared with two different average AgNW lengths: (1) 30 μm prepared with 10k PVP and (2) 183 μm synthesized with 1300k PVP and the ultimate nanowire density on the surface was adjusted to 0.24 g cm^−2^. After annealing the AgNW films at 250 °C for 30 min, the shorter AgNWs provided a sheet resistance of 100 Ω sq^−1^ and an optical transmission of 84% (at 550 nm) while the longer AgNWs demonstrated a sheet resistance and an optical transmission of 2 Ω sq^−1^ and 89% (at 550 nm), respectively, indicating a more percolated network structure, which is comparable to that in previous work.^46^ Annealing the AgNWs in an oven offers a facile and low-cost approach for improving the contact between the AgNWs compared to other welding techniques including chemical welding,^47,48^ laser welding,^49^ pressure welding,^50^ Joule heating^51^ and nano-soldering.^52^ The optical transmission graph of the AgNWs films is exhibited in Fig. S13.† A photograph demonstrating this transparent heater is shown in Fig. 10C. Putting together low sheet resistance coupled with high transmittance renders these films highly promising for optoelectronic applications requiring transparent conductive substrates.

Fig. 10A exhibits the temperature change of a film consisting of shorter AgNWs at 1, 3 and 5 V for 180 s, reaching a maximum temperature of 26, 40 and 64 °C, respectively. In contrast, as demonstrated in Fig. 10B, the films comprising longer AgNWs provided maximum temperatures of 38 °C (1 V), 125 °C (3 V) and 208 °C (5 V) thus indicating significantly a more efficient Joule heating effect. The corresponding thermal camera images for films made out of shorter and longer AgNWs at 5 V are exhibited in Fig. 10D and E. It is notable that longer AgNWs led to better optoelectronic and Joule heating performance. It can be stated that owing to its lower sheet resistance, the longer AgNW sample led to more electrical current through the sample which increased the temperature dramatically.

Compared to other AgNW-based transparent heater applications, the performance figures of which are collected and shown in Table 1, highlight the two distinct advantages of our work. The usage of ultralong AgNWs allows reaching high conductivity with very low AgNW loading on the surface, thus improving the overall transparency of the sample. This is a distinct advantage for any optoelectronic application, where a combination of high conductivity and high transparency over the visible range is required. Moreover, this work provided higher temperature (208 °C) with a relatively lower response time (70 s) and voltage (5 V). As shown in Table 1, previously reaching a temperature of over 200 °C required more than 10 times longer time when using AgNWs of 10–30 μm in diameter. This points to the second distinct advantage of our system, namely the reduced number of junctions with the ultra-long nanowires.

**Table 1:** Comparison of the current work with the transparent heaters in the literature

AgNW length (μm)	Optical transmission at 550 nm (%)	Sheet resistance (Ω sq^−1^)	Driving voltage (V)	Response time (s)	Max. temperature (°C)	Ref.
183	89	2	5	70	208	This work
20–30	84	12	7	45	128	10
—	>80	23	15	150	150	53
—	87	20	4	15	134	54
30	∼90	20	12	—	80	55
45–65	∼70	2.7	9	>300	155	56
10–30	84	4.3	4	>900	204	57
20–30	—	0.25	2	∼40	160	58
12	72	6	4	∼100	113	59
20–40	>80	10	7	60	<110	60
2–25	90	35	7	<200	55	61

## Conclusions

Ultralong AgNWs with an aspect ratio of >2000 were successfully obtained *via* optimization of the hydrothermal synthesis conditions and the nanowires enabled efficient thermal heaters. Major findings are summarized as follows:

(1) Optimum conditions for hydrothermal synthesis were evaluated as a reaction time of 24 h, reaction temperature of 160 °C, PVP MW of 1300k, PVP concentration of 125 mM, glucose concentration of 22.4 mM and CuCl_2_ concentration of 20 μM.

(2) It was demonstrated that changing a single synthesis parameter resulted in AgNWs with different average lengths. However, it was revealed that reaction temperature, PVP MW and PVP concentration have more significant influences on the nanowire length.

(3) Optimum conditions provided AgNWs with an average diameter of 207 nm, an average length of 234 μm, a maximum length of 397 μm and an aspect ratio of >1000.

(4) Replacing CuCl_2_ with FeCl_3_ provided thinner nanowires with an average diameter of 131 nm, which in turn led to an aspect ratio of >2000.

(5) Ultralong AgNWs enabled superior optoelectronic and thermal heater properties compared to those in previous studies.

As a conclusion, optimization of hydrothermal synthesis parameters provided ultra-long AgNWs with a high aspect ratio. In addition, when assessing the performance of a transparent thermal heater, two distinct advantages of using ultralong AgNWs were found. First, employing these AgNWs allowed reducing the surface loading of a conductive nanomaterial leading thus to improved transparency over the visible wavelength range as well as sustainability benefits. Secondly, the reduced number of AgNW junctions improved the efficiency of the Joule heating process enabling to reach high temperature in a much shorter time as compared to thermal heaters made out of shorter AgNWs.

## Author contributions

Fevzihan Basarir: funding acquisition, conceptualization, methodology, writing – original draft. Swarnalok De: formal analysis, methodology, writing – review & editing. Hamidreza Daghigh Shirazi: visualization, writing – review & editing. Jaana Vapaavuori: funding acquisition, supervision, project administration, writing – review & editing.

## Conflicts of interest

The authors declare no conflict of interest.

## Supplementary Material

NA-004-D2NA00560C-s001
