# Supplementary material for: Ultra-long silver nanowires prepared via hydrothermal synthesis enable efficient transparent heaters
Source: Nanoscale Adv. 2022 Aug 29;4(20):4410–7. doi: 10.1039/d2na00560c (PMC9552902; doi:10.1039/d2na00560c)
Supplement: NA-004-D2NA00560C-s001 [file NA-004-D2NA00560C-s001.pdf]

Electronic Supplementary Material (ESI) for Nanoscale Advances.
This journal is © The Royal Society of Chemistry 2022

SUPPORTING INFORMATION

Ultra-long silver nanowires prepared via hydrothermal synthesis enable efficient transparent heaters

Fevzihan Basarir,[a] Swarnalok De,[a] Hamidreza Daghigh Shirazi [a] and Jaana Vapaavuori *[a]

Department of Chemistry and Materials Science, Aalto University, P.O. Box 16100, FI-00076 AALTO, Finland

***Corresponding Author**

Prof. Jaana Vapaavuori

E-mail: jaana.vapaavuori@aalto.fi

Table S1. Summary of the AgNW synthesis conditions and the nanowire length

Sample	$AgNO_3$ concentration (mM)	Reaction time (hour)	Reaction temperature (°C)	PVP MW (g/mol)	PVP concentration (mM)	Glucose concentration (mM)	$CuCl_2$ concentration (µM)	Max. length (µm)	Average length (µm)
1	50	8	160	360K	75	16.8	20	47	18
2	50	16	160	360K	75	16.8	20	127	58
3	50	24	160	360K	75	16.8	20	253	105
4	50	32	160	360K	75	16.8	20	273	106
5	50	24	150	360K	75	16.8	20	70	27
6	50	24	160	360K	75	16.8	20	253	105
7	50	24	170	360K	75	16.8	20	160	69
8	50	24	180	360K	75	16.8	20	51	22
9	50	24	160	10K	75	16.8	20	78	30
10	50	24	160	55K	75	16.8	20	141	63
11	50	24	160	360K	75	16.8	20	253	105
12	50	24	160	1300K	75	16.8	20	327	183
13	50	24	160	360K	50	16.8	20	196	70
14	50	24	160	360K	75	16.8	20	253	105
15	50	24	160	360K	100	16.8	20	362	190
16	50	24	160	360K	125	16.8	20	363	197
17	50	24	160	360K	75	5.6	20	114	46
18	50	24	160	360K	75	11.2	20	199	77
19	50	24	160	360K	75	16.8	20	253	105
20	50	24	160	360K	75	22.4	20	255	119
21	50	24	160	360K	75	16.8	10	113	29
22	50	24	160	360K	75	16.8	20	183	57
23	50	24	160	360K	75	16.8	50	244	105
24	50	24	160	360K	75	16.8	100	253	106

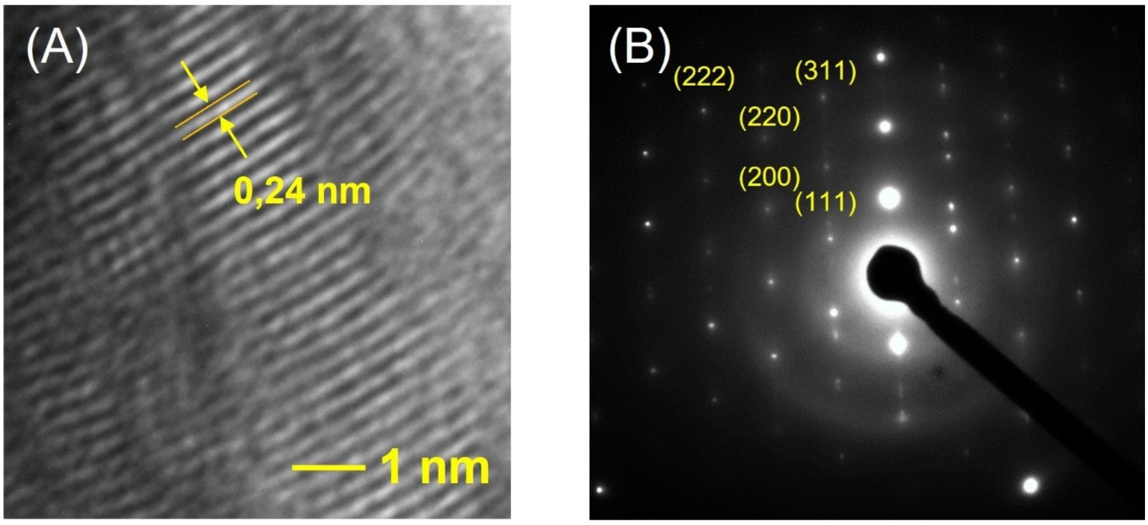

Figure S1. SAED pattern and electron diffraction pattern of the AgNWs.

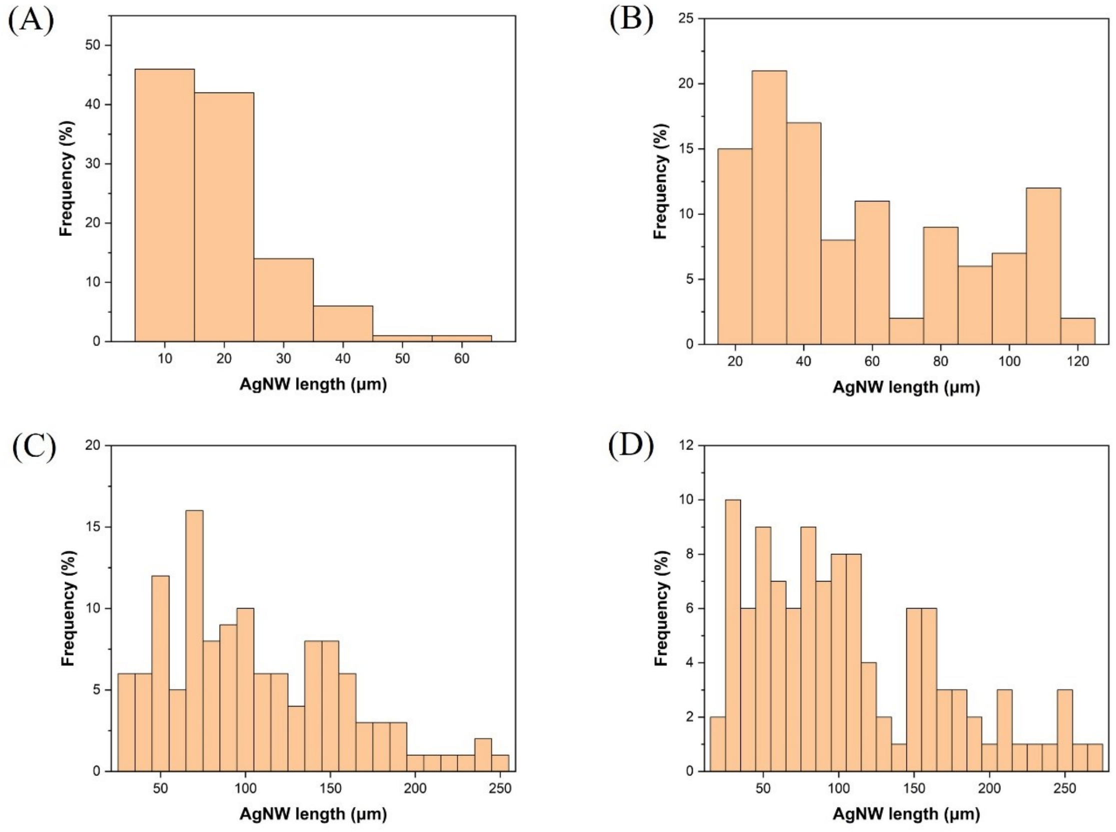

Figure S2. Length distribution of the AgNWs synthesized at (A) 8 hours, (B) 16 hours, (C) 24 hours and (D) 32 hours.

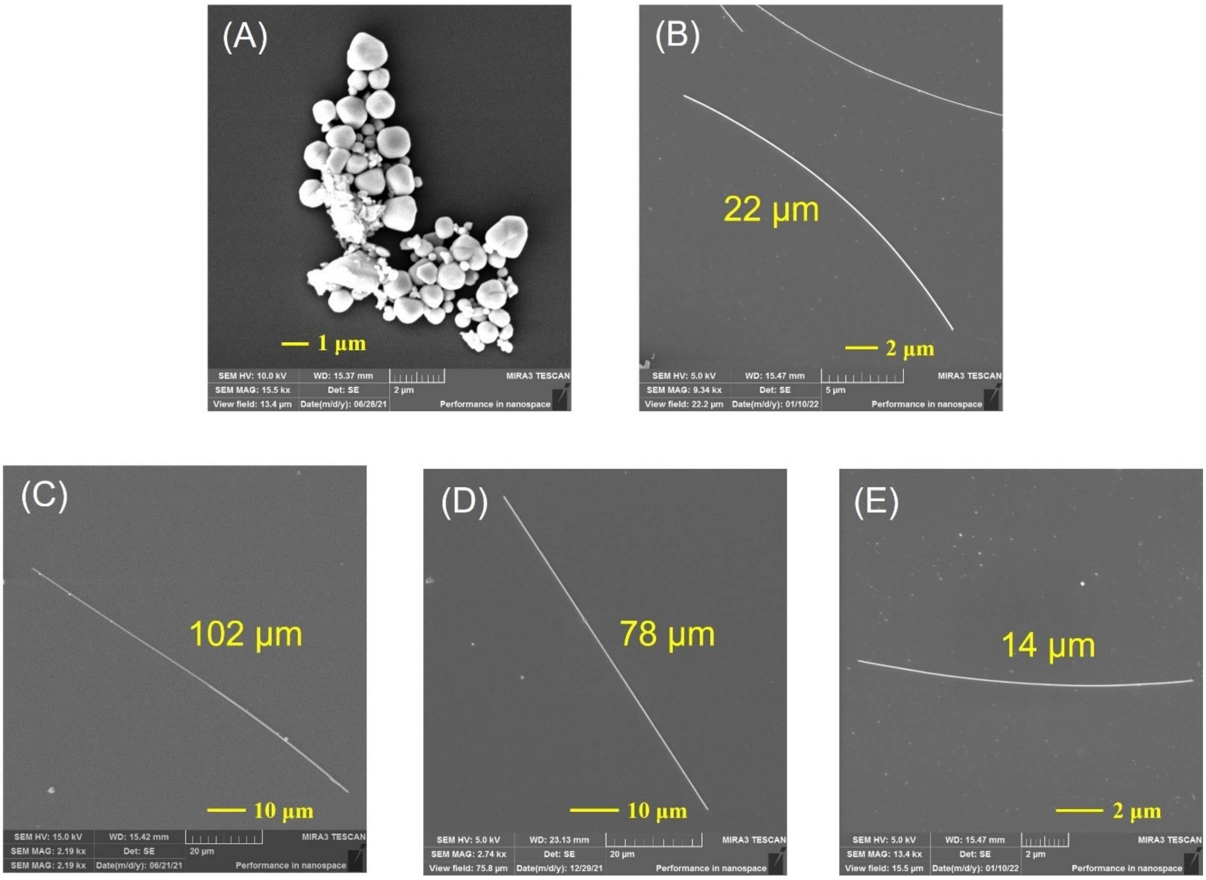

Figure S3. SEM images of the AgNWs synthesized at (A) 140 ^{o}C, (B) 150 ^{o}C, (C) 160 ^{o}C, (D) 170 ^{o}C and (E) 180 ^{o}C.

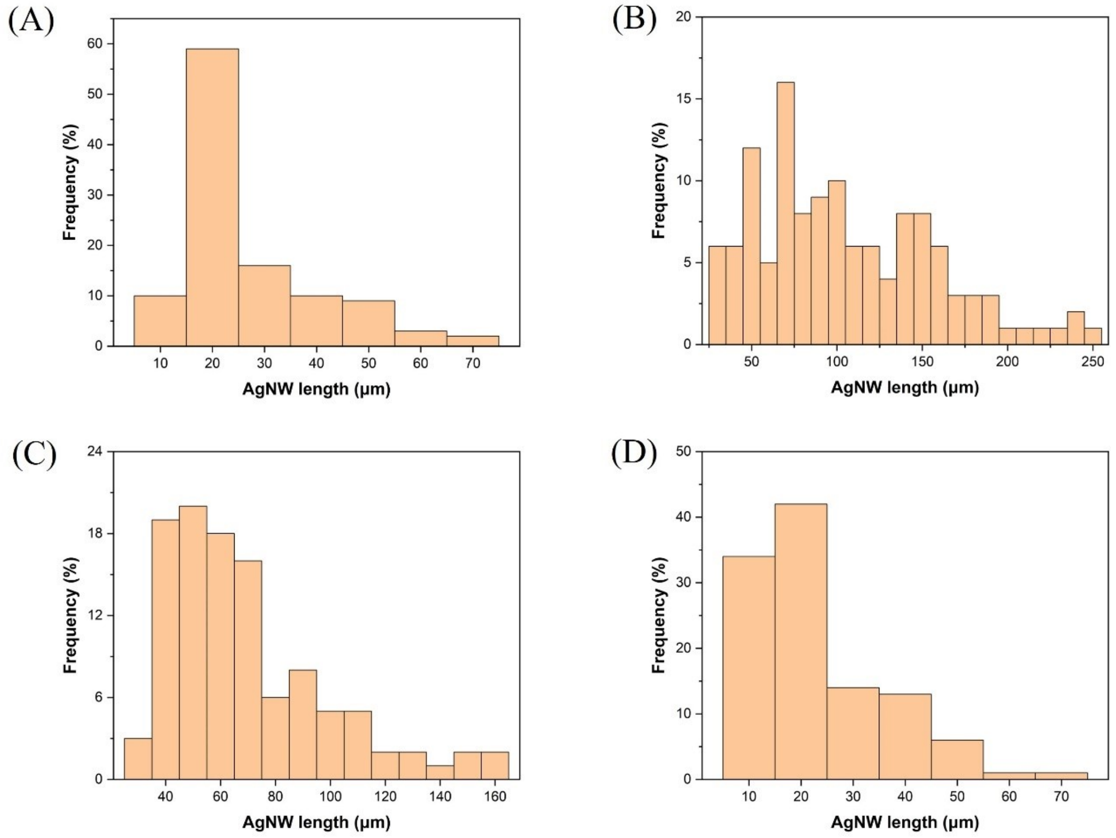

Figure S4. Length distribution of the AgNWs synthesized at (A) 150 °C, (B) 160 °C, (C) 170 °C and (D) 180 °C.

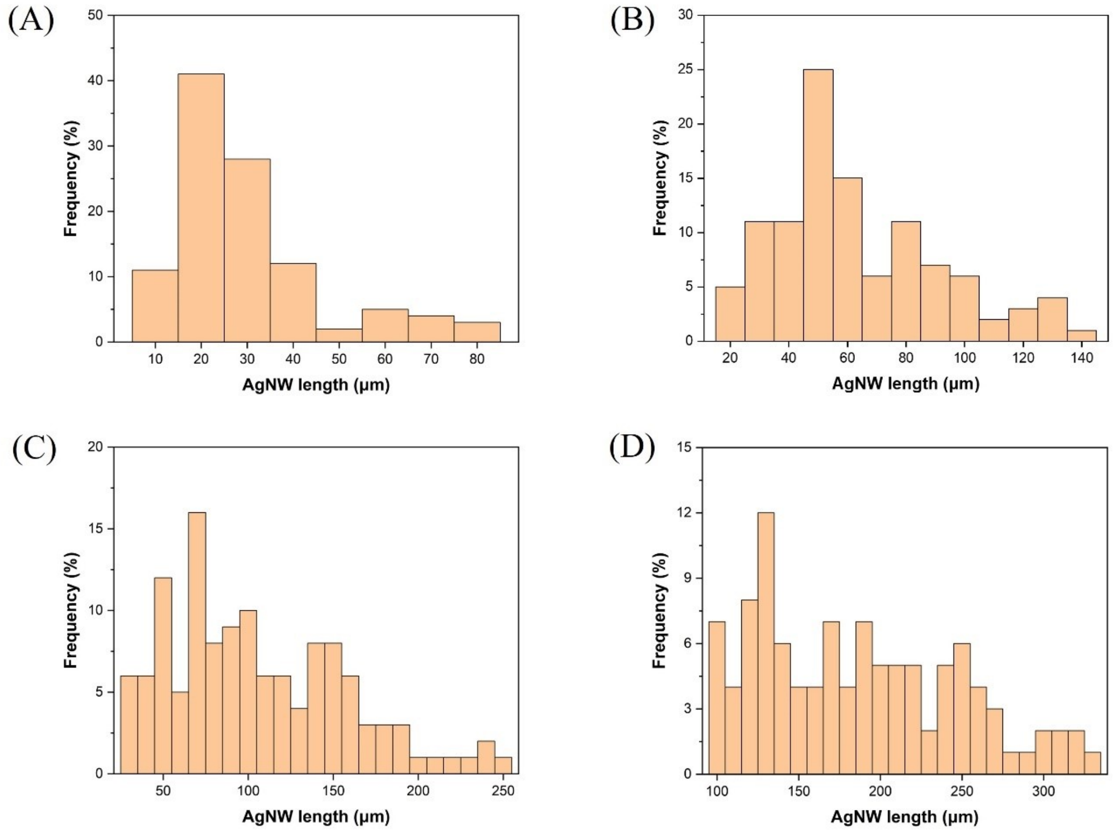

Figure S5. Length distribution of the AgNWs synthesized with PVP MW of (A) 10K, (B) 55K, (C) 360K and (D) 1300 K.

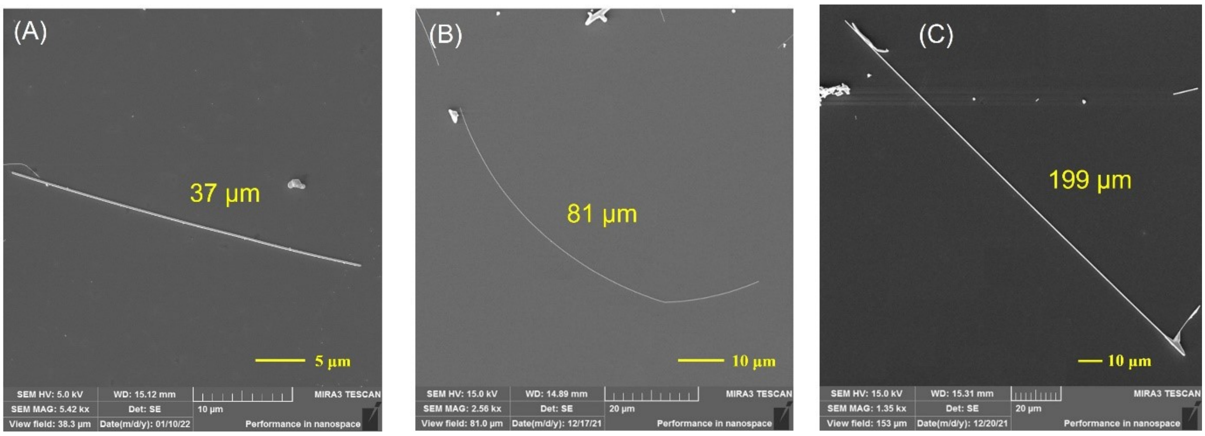

Figure S6. SEM images of the AgNWs synthesized with PVP MW of (A) 10K, (B) 55K and (C) 1300K.

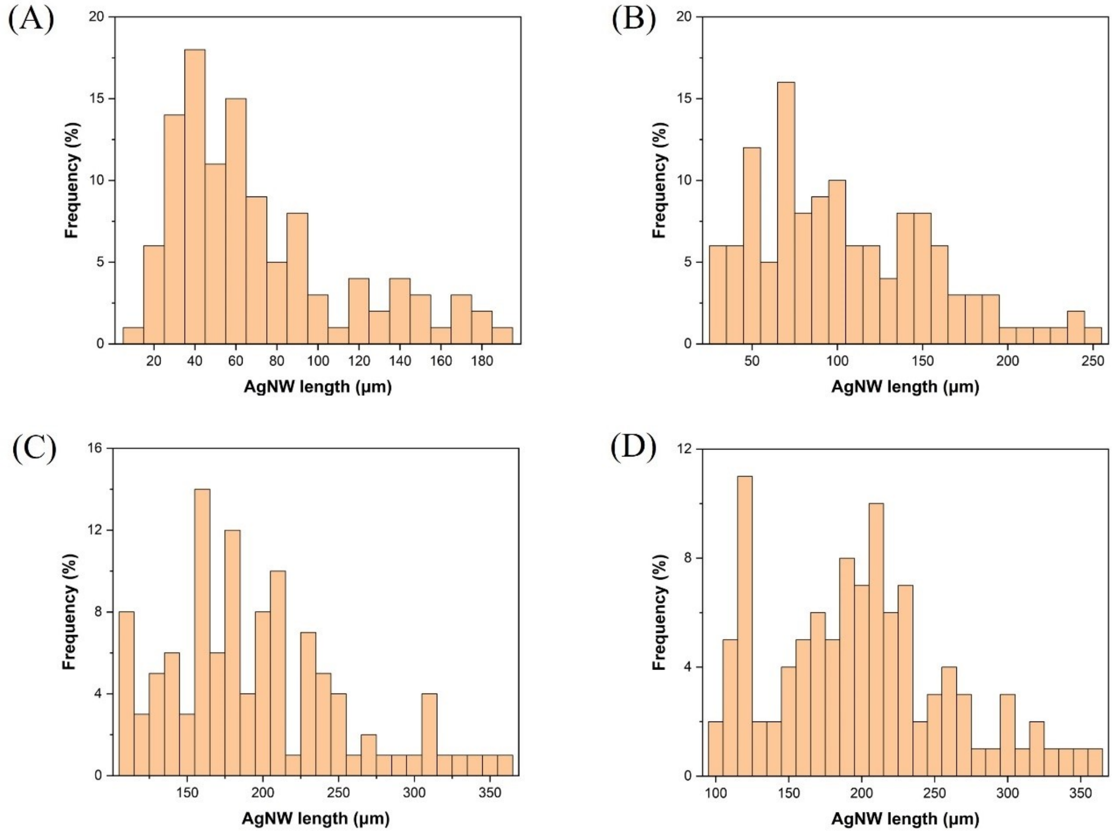

Figure S7. Length distribution of the AgNWs synthesized with PVP concentration of (A) 50 mM, (B) 75 mM, (C) 100 mM and (D) 125 mM.

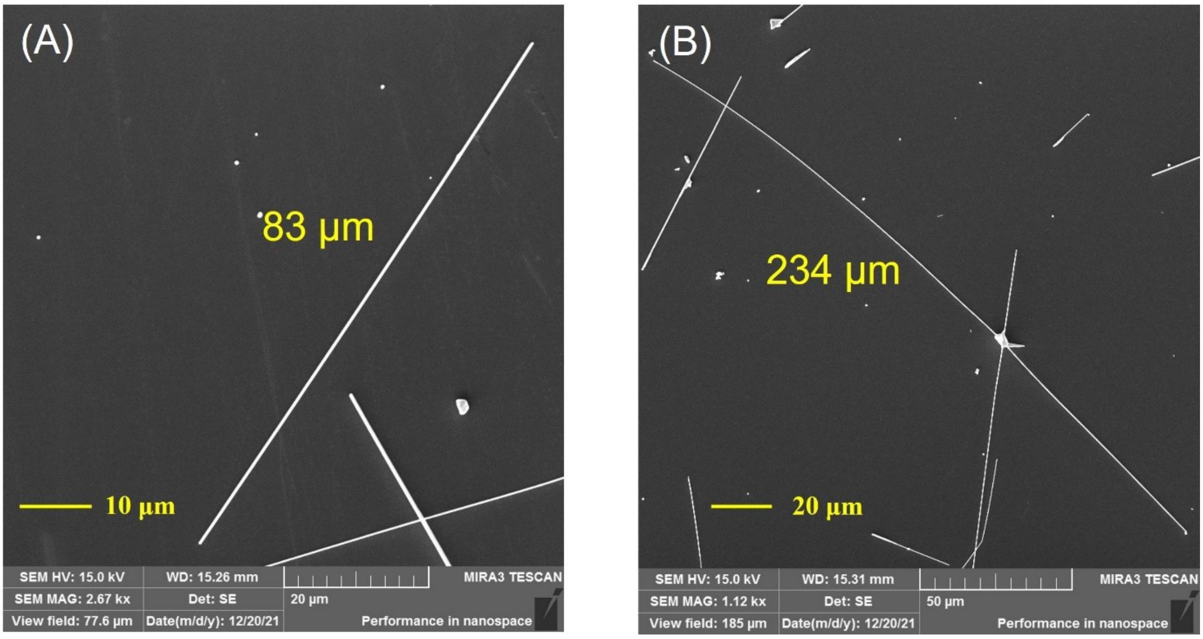

Figure S8. SEM images of the AgNWs synthesized with PVP (360K) concentration of (A) 50 and (B) 100 mM.

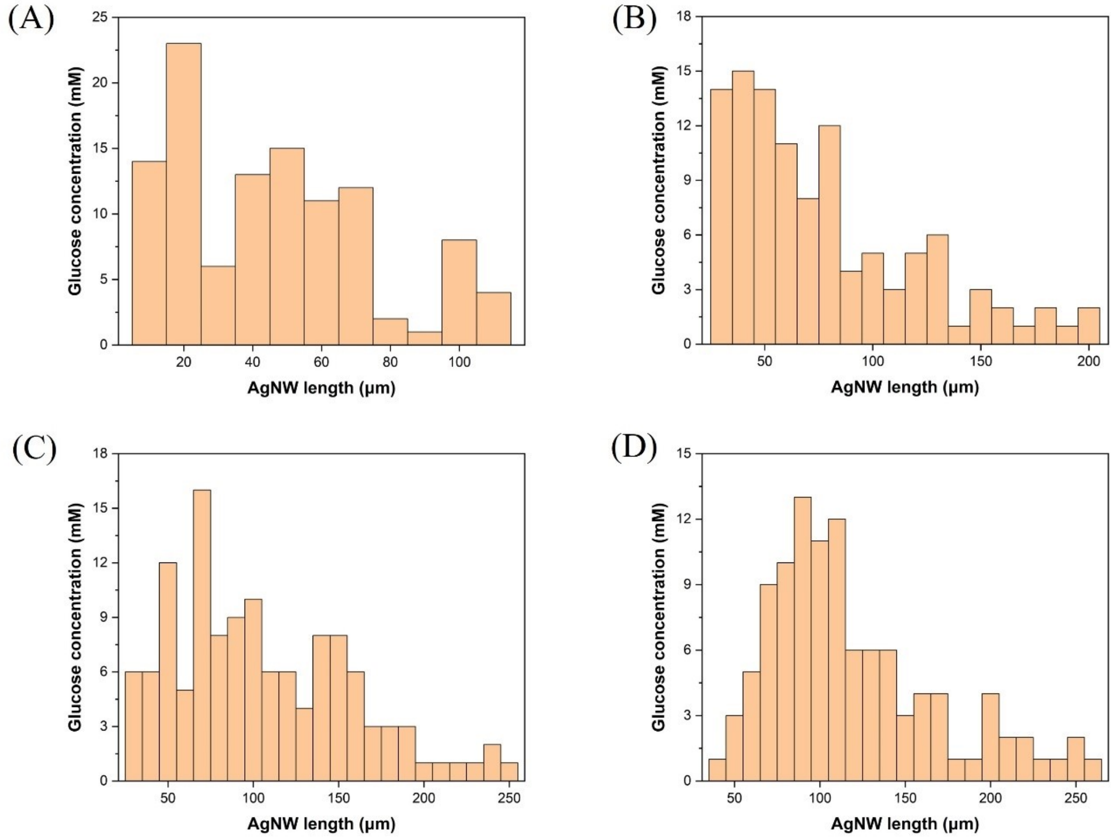

Figure S9. Length distribution of the AgNWs synthesized with glucose concentration of (A) 5.6 mM, (B) 11.2 mM, (C) 16.8 mM and (D) 22.4 mM.

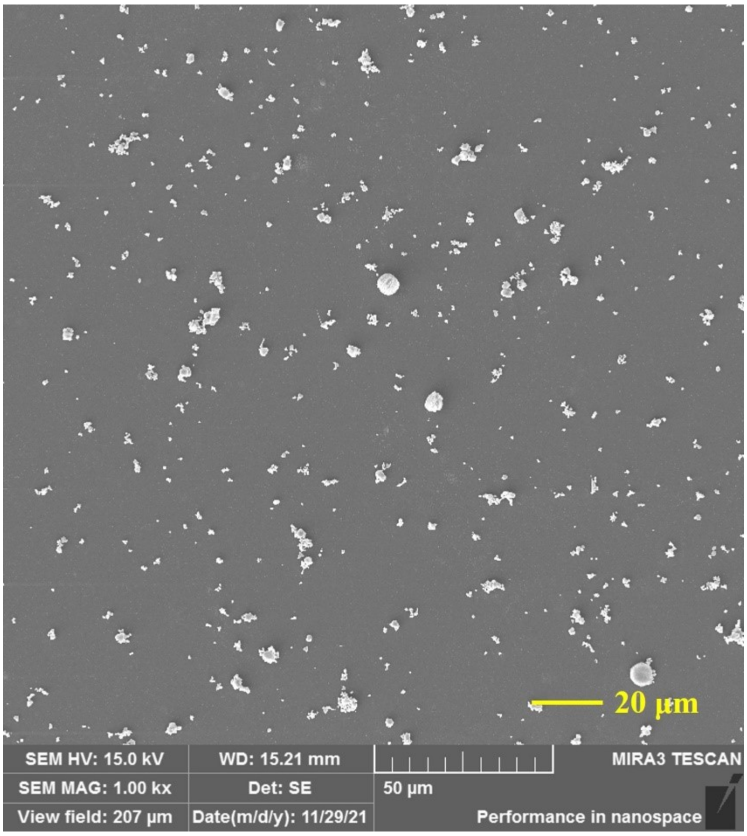

Figure S10. SEM images of the AgNWs synthesized with glucose concentration of 2.8 mM.

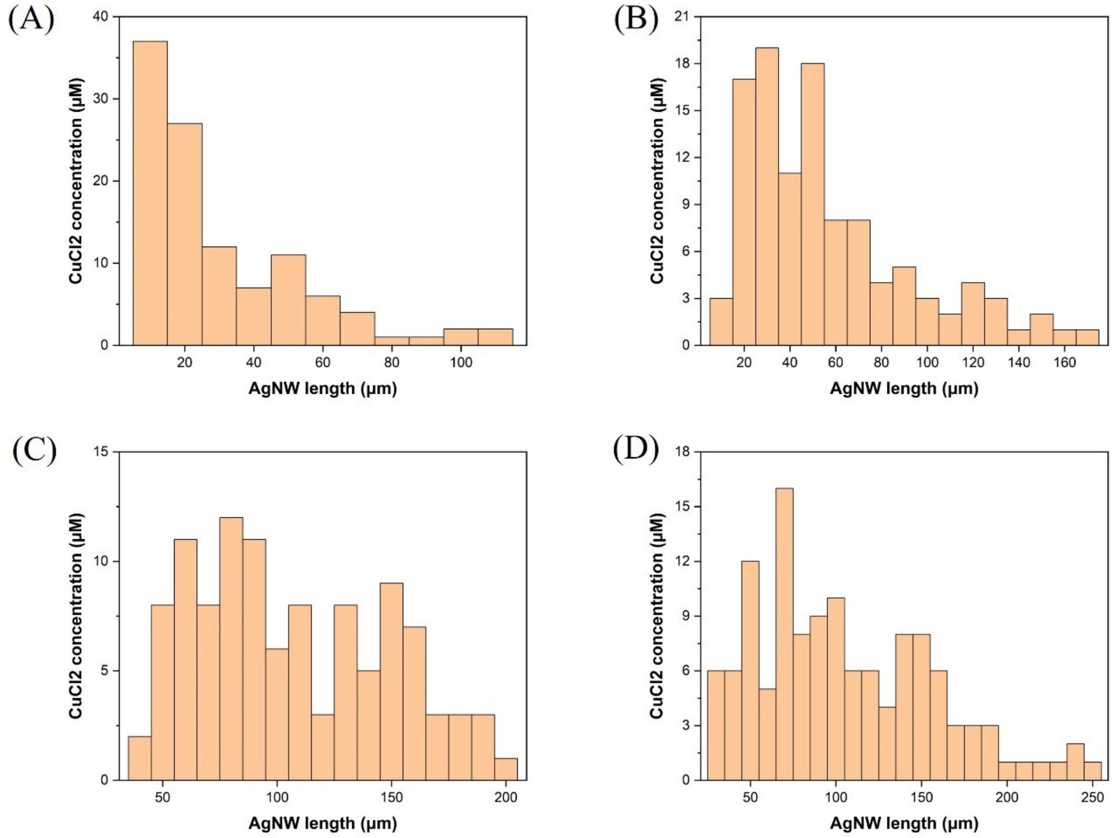

Figure S11. Length distribution of the AgNWs synthesized with $CuCl_2$ concentration of (A) 2 μM, (B) 4 μM, (C) 10 μM and (D) 20 μM.

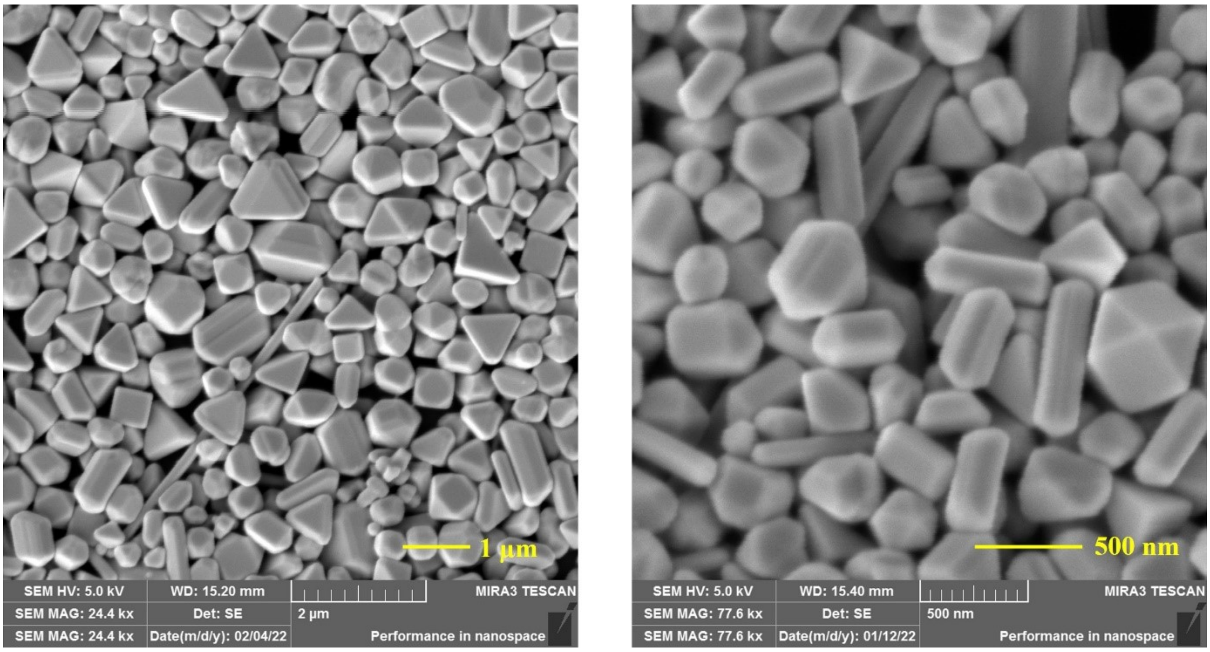

Figure S12. SEM images of the byproducts of the AgNWs synthesis with $CuCl_2$ (A) and $FeCl_3$ (B).

Table S2. Standard Reduction Potentials of the metal ions

$Cu^{2+} + e^-$	$\longrightarrow$	Cu^+	+0.16 V
$Fe^{3+} + e^-$	$\longrightarrow$	Fe^{2+}	+0.77 V
Na^+ + e-	$\longrightarrow$	Na(s)	-2.71 V

Reference: Table of Standard Electrode Potentials (gsu.edu)

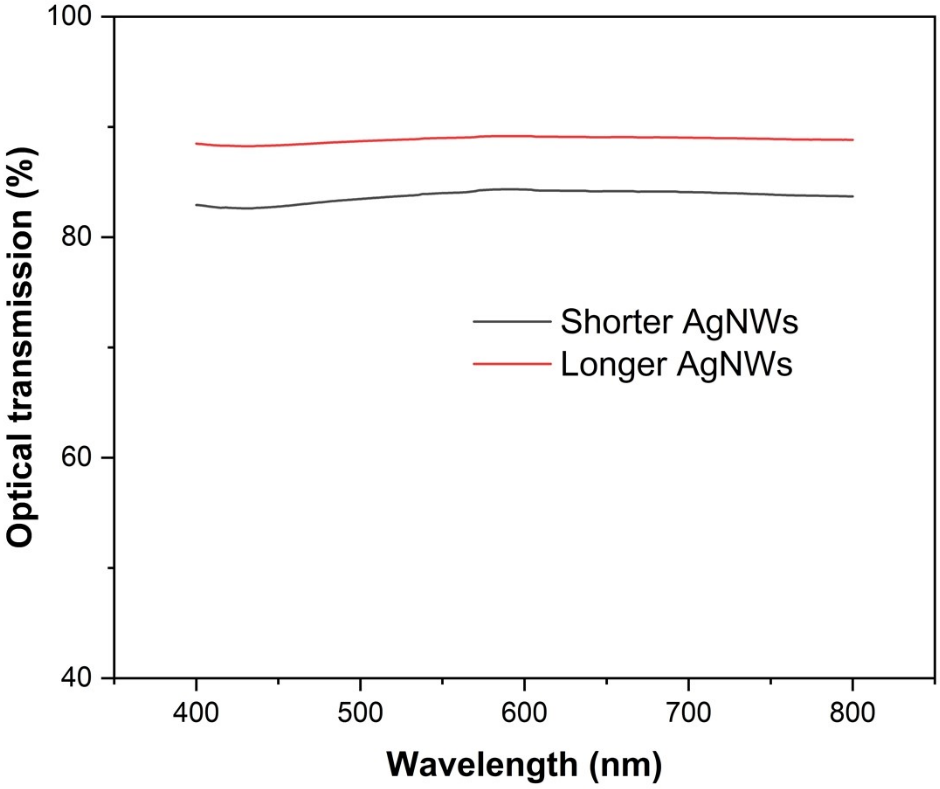

Figure S13. Optical transmission of AgNW films with a density of 0.24 gr/cm^2.
